# A comparison of three commercial IMRT treatment planning systems for selected pediatric cases

**DOI:** 10.1120/jacmp.v13i2.3742

**Published:** 2012-03-08

**Authors:** Ismail Eldesoky, Ehab M. Attalla, Wael M. Elshemey, Mohamed S. Zaghloul

**Affiliations:** ^1^ Children's Cancer Hospital Cairo; ^2^ National Cancer Institute Cairo University Cairo; ^3^ Department of Biophysics, Faculty of Science Cairo University Cairo Egypt

**Keywords:** KonRad, XiO, Prowess, IMRT

## Abstract

This work aimed at evaluating the performance of three different intensity‐modulated radiotherapy (IMRT) treatment planning systems (TPSs) — KonRad, XiO and Prowess — for selected pediatric cases. For this study, 11 pediatric patients with different types of brain, orbit, head and neck cancer were selected. Clinical step‐and‐shoot IMRT treatment plans were designed for delivery on a Siemens ONCOR accelerator with 82‐leaf multileaf collimators (MLCs). Plans were optimized to achieve the same clinical objectives by applying the same beam energy and the same number and direction of beams. The analysis of performance was based on isodose distributions, dose‐volume histograms (DVHs) for planning target volume (PTV), the relevant organs at risk (OARs), as well as mean dose $\left(D_{\text{mean}}\right)$, maximum dose $\left(D_{\text{max}}\right)$, 95% dose $\left(D_{95}\right)$, volume of patient receiving 2 and 5 Gy, total number of segments, monitor units per segment (MU/Segment), and the number of MU/cGy. Treatment delivery time and conformation number were two other evaluation parameters that were considered in this study. Collectively, the Prowess and KonRad plans showed a significant reduction in the number of MUs that varied between 1.8% and 61.5% (p−value=0.001) for the different cases, compared to XiO. This was reflected in shorter treatment delivery times. The percentage volumes of each patient receiving 2 Gy and 5 Gy were compared for the three TPSs. The general trend was that KonRad had the highest percentage volume, Prowess showed the lowest (p−value=0.0001). The KonRad achieved better conformality than both of XiO and Prowess. Based on the present results, the three treatment planning systems were efficient in IMRT, yet XiO showed the lowest performance. The three TPSs achieved the treatment goals according to the internationally approved standards.

## I. INTRODUCTION

A tremendous evolution in treatment process occurred in recent years. This allowed the delivery of the desired radiation dose distribution to target tissue, while delivering an acceptable radiation dose to the surrounding normal tissues with greater dose gradients and tighter margins. The treatment planning system (TPS) has a central role in the application of IMRT technique. A modern TPS has more sophisticated calculation algorithms, providing more accurate dose calculation capabilities, especially for the small beams associated with IMRT delivery techniques. Automated optimization routines used in conjunction with inverse planning are available to help define the multileaf collimator delivery configurations.^(^ ^1^ ^,^ ^2^ ^)^

Reported comparisons between TPSs included different parameters such as the accuracy of calculations, implementation and commissioning algorithms used in the optimization process, and the clinical functionality of the plan. Haslam et al.^(3)^ compared the isocenter dose calculated by each of a commercial IMRT treatment planning system and an independent monitor unit verification calculation software in order to estimate the tolerance for monitor unit calculations. Pflugfelder et al.^(4)^ proposed an improved optimization algorithm that could reach the same objective function value six times faster than commercial ones. Petric et al.^(5)^ compared BrainScan TPS (BrainLAB, Feldkirchen, Germany) to Eclipse/Helios TPS (Varian Medical Systems, Palo Alto, CA) in terms of implementation and commissioning, dose optimization, and plan assessment. Their results referred to an inadequacy of the Eclipse TPS to accurately calculate dose for highly modulated fields. Comparison between TPSs based on the evaluation of clinical functionality of the plan was rare especially in the pediatric field.^(6)^

One important factor in the comparison of TPSs is the time of radiation delivery to patients. Cancer patients may have difficulties lying on the treatment couch for long periods of time during the radiation delivery. Shortening the IMRT treatment time decreases the risk that patients involuntarily move during radiation therapy. It also minimizes the risk of decreased tumor cell killing potentially associated with delivery times in the range of 15ñ45 min.^(7)^ Shortening the IMRT treatment time is highly desirable, especially for pediatric patients.^(8)^

Although IMRT in pediatric cases is definitely more complicated than in adults, only few studies comparing different commercial planning systems for IMRT in pediatric patients do exist. Therefore, the present study is concerned with introducing an experience with some pediatric indications that were chosen to represent common cases seen in the brain, orbit, and head and neck regions.

The objective of this work is to evaluate the performance of three different IMRT treatment planning systems (TPSs), Siemens KonRad version 2.2.23, Elekta XiO version 4.4, and Prowess Panther version 5, for brain, orbit, and head and neck cancer patients.

## II. MATERIALS AND METHODS

The CT image sets for 11 pediatric patients, presenting different types of brain, orbit, head and neck cancers, were sent through the department network system (LANTIS Oncology Management System) to three different (TPSs), Siemens KonRad version 2.2.23 Siemens, Malvern, PA), Elekta XiO version 4.4 (Elekta CMS, Maryland Heights, MO), and Prowess Panther version 5 (Prowess inc., Concord, CA). In Table 1, a summary of the diagnosis, dose prescriptions, and clinical objectives (CObj) for organs at risk (OAR) is presented. All of the dose constraints reported here are specific to pediatric cases and are more restrictive than those used for adults.^(^ ^9^ ^,^ ^10^ ^)^

**Table 1:** A summary of the diagnosis, dose prescriptions, and clinical objectives (CObj) for organs at risk (OAR) of the investigated cases.

	*Case 1*	
Low‐grade Glioma	Diagnosis
Total=54Gy,1.8Gy/fraction	Radiotherapy dose prescription
$\text{PTV}=264\;{\text{cm}}^{3}$	Target volumes
Eye mean dose <40Gy, Cochlea mean dose <40Gy Brain stem maximum dose <45Gy	Organs at risk dose objectives
7 fields with gantry angles: 0, 51, 103, 154, 206, 257, 308	Beam arrangement
	*Case 2*	
Parotid Carcinoma	Diagnosis
Total=64.8 Gy, 1.8 Gy/fraction	Radiotherapy dose prescription
$\text{PTV}=232\;{\text{cm}}^{3}$	Target volumes
Spinal cord maximum dose <45Gy, Parotid mean dose <26Gy, Brain stem maximum dose <45Gy	Organs at risk dose objectives
6 fields with gantry angles: 27, 129, 180, 231, 282, 333	Beam arrangement
	*Case 3*	
Pituitary Adenoma	Diagnosis
Total=50 Gy, 2 Gy/fraction	Radiotherapy dose prescription
$\text{PTV}=72.8\;{\text{cm}}^{3}$	Target volumes
Eye mean dose <40Gy, Brain stem maximum dose <45Gy	Organs at risk dose objectives
7 fields with gantry angles: 0, 51, 103, 154, 206, 257, 308	Beam arrangement
	*Case 4*	
Retinoblastoma	Diagnosis
Total=45 Gy, 1.8 Gy/fraction	Radiotherapy dose prescription
$\text{PTV}=60.9\;{\text{cm}}^{3}$	Target volumes
Eye mean dose <40Gy, Lacrimal gland maximum dose <41Gy, Brain stem maximum dose <45Gy	Organs at risk dose objectives
8 fields with gantry angles: 27, 78, 231, 282, 333, with couch angle zero, and 270,315,45 with couch angle 90	Beam arrangement
	Case 5	
Parotid carcinoma	Diagnosis
Total=64.8 Gy, 1.8 Gy/fraction	Radiotherapy dose prescription
$\text{PTV}=94.7\;{\text{cm}}^{3}$	Target volumes
Spinal cord maximum dose <45Gy Brain stem maximum dose <45Gy	Organs at risk dose objectives
6 fields with gantry angles: 27, 78, 129, 180, 220, 333	Beam arrangement
	*Case 6*	
High‐risk Medulloblastoma (Posterior fossa)	Diagnosis
Total=19.8 Gy, 1.8 Gy/fraction. (Precribed dose is only the boost dose which was used in the IMRT plan.)	Radiotherapy dose prescription
$\text{PTV}=272\;{\text{cm}}^{3}$	Target volumes
Eye mean dose <40Gy, Cochlea mean dose <40Gy Optic nerve maximum dose <50Gy	Organs at risk dose objectives
7 fields with gantry angles: 27, 78, 129, 180, 231, 282,333	Beam arrangement
	*Case 7*	
Low‐risk Medulloblastoma (Posterior fossa)	Diagnosis
Total=32.4 Gy, 1.8 Gy/fraction (Prescribed dose is only the boost dose which was used in the IMRT plan.)	Radiotherapy dose prescription
$\text{PTV}=197\;{\text{cm}}^{3}$	Target volumes
Pituitary mean dose <25Gy, Cochlea mean dose <40Gy Brain stem maximum dose <45Gy	Organs at risk dose objectives
7 fields with gantry angles: 0, 51, 103, 154, 206, 257, 308	Beam arrangement
7 fields with gantry angles: 0, 51, 103, 154, 206, 257, 308	Beam arrangement
	*Case 8*	
Nasolaboil Rhabdomyosarcoma	Diagnosis
Total=36 Gy, 1.8 Gy/fraction	Radiotherapy dose prescription
$\text{PTV}=36.6\;{\text{cm}}^{3}$	Target volumes
Eye mean dose <40Gy Optic nerve maximum dose <50Gy	Organs at risk dose objectives
7 fields with gantry angles: 0, 40, 80, 120, 240, 280, 320	Beam arrangement
	*Case 9*	
Rhabdomyosarcoma(RMS)	Diagnosis
Total=50.4 Gy, 1.8 Gy/fraction	Radiotherapy dose prescription
$\text{PTV}=942.6\;{\text{cm}}^{3}$	Target volumes
Eye mean dose <40Gy, Parotid mean dose <26Gy Brain stem maximum dose <45Gy	Organs at risk dose objectives
7 fields with gantry angles: 27, 78, 129, 180, 231, 282, 333	Beam arrangement
	*Case 10*	
Ependymoma	Diagnosis
Total=59.4 Gy, 1.8 Gy/fraction	Radiotherapy dose prescription
$\text{PTV}=262.8\;{\text{cm}}^{3}$	Target volumes
Eye mean dose <40Gy, Cochlea mean dose <40Gy Brain stem maximum dose <45Gy	Organs at risk dose objectives
7 fields with gantry angles: 27, 78, 129, 180, 231, 282, 333	Beam arrangement
	*Case 11*	
Maxillary Sarcoma	Diagnosis
Total=60 Gy, 2 Gy/fraction	Radiotherapy dose prescription
$\text{PTV}=259.9\;{\text{cm}}^{3}$	Target volumes
Eye mean dose <40Gy, Parotid, mean dose <26Gy Brain stem maximum dose <45Gy	Organs at risk dose objectives
9 fields with gantry angles: 0, 40, 80, 120, 160, 200, 240, 280, 320	Beam arrangement

The clinical IMRT treatment plans were designed using the three treatment planning systems as step‐and‐shoot IMRT plans for delivery on the same machine, a Siemens ONCOR accelerator (Siemens, Malvern, PA) with an 82‐leaf MLC. Using the same machine nutralizes any limitation, due to machine configuration such as the leaf width or radiation leakage. On each of the three planning systems, three objectives were fulfilled before the plan was accepted: i) target coverage heterogeneity within +7% and −5% of the prescribed dose (according to International Commission on Radiation Units and Measurements (ICRU)), ii) OAR sparing to at least the limits stated in Table 1, and iii) sparing of healthy tissue (the CT dataset patient volume minus the volume of the largest target).^(11)^ The number of fields and the beam geometry were fixed in order to avoid variability in the results due to different beam arrangements.

Both KonRad and XiO treatment planning systems have optimization engines which rely on physical optimization. The dose calculation was performed using either pencil beam (PB) in the case of KonRad,^(^ ^12^ ^,^ ^13^ ^)^ or superposition algorithm in the case of XiO. Both treatment planning systems used the dose volume constraints and minimumñmaximum dose constraints.^(14)^

Prowess uses the direct aperture optimization (DAO), so it has convolution/superposition dose calculation engine, which includes all the delivery constraints within the optimization process, as well as the weights of the individual aperture shapes. There is no conversion from fluence maps to aperture shapes.^(15)^

The number of intensity levels used by the three systems to discretize individual beam fluence was determined manually in order to achieve the clinical goals with the fewest number of segments.

Special care was taken during this pre‐optimization phase in order to ensure that an adequate number of calculation points were defined within each structure, as this would influence the optimization results.

### A. Evaluation tools

The analysis was based on isodose distributions and on dose‐volume histograms (DVHs) for planning target volume (PTV) and the relevant OARs, as well as mean dose, maximum dose, $D_{95}$ (dose to 95% of the PTV).^(16)^ Volumes receiving 2 Gy and 5 Gy were calculated and compared. Also the treatment delivery time, total number of segments, MU/segment, and the number of MU/cGy were investigated.^(17)^

Conformation number (CN) as described by Riet et al.^(18)^ was used because it took into account irradiation of the target volume and irradiation of healthy tissues. This number was defined as follows:

(1) $$\text{CN}={\text{TV}}_{\text{RI}}/\text{TV}*{\text{TV}}_{\text{RI}}/V_{\text{RI}}$$

where CN=conformationnumber, ${\text{TV}}_{\text{RI}}=$ target volume covered by the reference isodose, TV=targetvolume, and $V_{\text{RI}}=$ volume of the reference isodose. The used reference isodose was the isodose 95% of the prescribed dose (according to the ICRU). The first fraction of this equation defines the quality of coverage of the target (local control), while the second fraction defines the volume of healthy tissue receiving a dose greater than or equal to the prescribed reference dose. The CN ranges from 0 to 1, where 1 is the ideal value.

Analysis of variance (ANOVA) test was performed as a statistical model used to study the significance level all through the data, and a p‐value less than 0.05 was considered statistically significant. In the study, alpha (α)=0.05.

Finally, the delivered doses had a complex, nonintuitive relationship to the number of monitor units. It was also impossible to predict the exact combination of field segments or the leaf motion patterns. Therefore, all the IMRT plans, which were performed using the MLC for the production of fluence modulations, should establish a precise and reliable method for the dosimetric verification of IMRT plans.^(19)^ Since a verification of dose distributions within a real patient was not possible, the phantom substitution method was often used.^(20)^

## III. RESULTS & DISCUSSION

**Figure 1:**
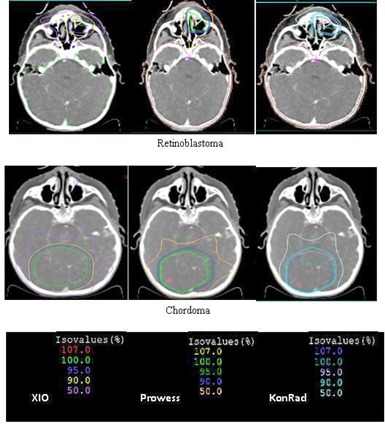
Dose distributions for some of the investigated cases from the three TPSs: KonRad (results in the right column), Prowess (middle column), XiO (left column).

Figure (1) shows the dose distributions obtained from the three TPSs (XiO, KonRad, and Prowess) for some of the investigated cases. The dose distributions are presented as a color isodose lines overlaid on the transverse CT slice through the isocenter. Comparing the dose distribution through the patient volume (cut‐by‐cut) makes it possible to qualitatively analyze the different degrees of conformal avoidance, the extension of the low‐dose areas, the degree of uniformity of doses within the PTVs, and the potential presence of hot spots. The dose distributions obtained from the three TPSs are found to be similar with minor differences. All of the plans achieve similar coverage of the PTVs. It is also demonstrated that although the dose distributions are similar, the relative beam weights of the fields can be different, depending on the treatment planning system.

**Figure 2:**
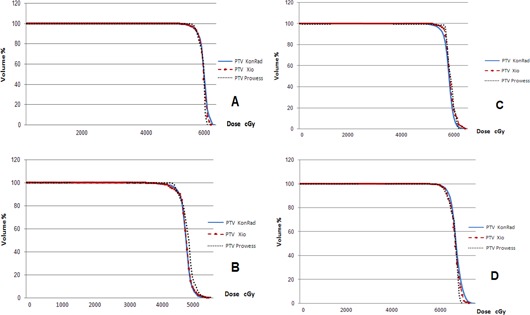
A comparison of the DVHs of some of the investigated cases for the PTVs from the three TPSs: KonRad (solid lines), XiO (dashed lines), Prowess (dotted lines).

Figure (2) presents a comparison of the DVHs of some of the investigated cases for the PTVs from the three TPSs. Such a comparison provides more quantitative results compared to the qualitative comparison of the dose distributions. All plans of the TPSs achieve similar PTV coverage. In most instances, a slightly steeper dose gradient in the case of the Prowess plans is noticed. Although the PTV coverage is similar for the three TPSs, the DVHs for OARs differ between the plans generated by the different planning systems.

**Figure 3:**
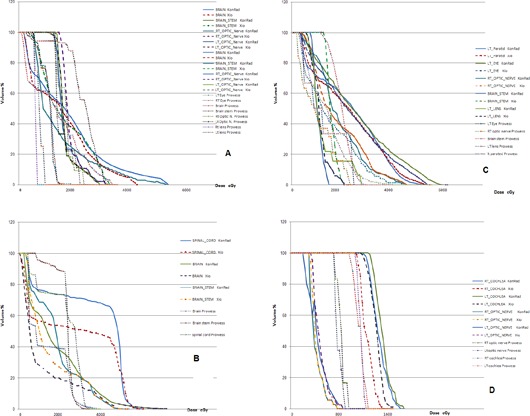
A comparison of the DVHs for the OARs from the KonRad (solid line), Prowess (gradient line), and XiO (dashed line) systems for some of the investigated cases.

Figure (3) shows the DVHs calculated by each of the three TPSs for some of the investigated cases. Several remarks can be drawn from each case. For patient 1, the three planning systems can easily reach the clinical objectives for the optic nerve. A better sparing of both cochleas is obtained by Prowess while maintaining the same improvement in both of the optic nerves. This reflects an ability of the Prowess TPS to achieve high conformality than KonRad and XiO. In case of patient 2, the objectives selected for the eye (that was partially included in the target) are fulfilled by the three TPSs. The high number of OARs in patient 3 does not cause any problem and the clinical objectives are respected, but it comes at the expense of MU especially in case of XiO. For patient 4, both XiO and Prowess show a better sparing in the entire OARs than KonRad. In patient 5, on average, all of the clinical objectives are fulfilled, despite the fact that for the maximum dose of most of the OARs, KonRad shows minor violations. In patient 6, the DVHs of Prowess for left parotid, left eye, show minor differences between the three TPSs, while the KonRad plan shows improved sparing of the brain stem. In the plan of patient 7, all of the clinical objectives are achieved with the minor exception of the parotid where the mean dose was 26.6 Gy instead of 26 Gy in the KonRad plan. Doses greater than 50 Gy are observed for the brain stem, exceeding the tolerance, with the KonRad plan in case of patient 8 because it fails to achieve a sharp dose falloff outside the target volume. For patient 9, XiO is able to reach the objectives for the spinal cord and brain stem associated with a consistently better management of the maximum dose and a relatively low‐dose bath.

Tables 2(a) and (b) show the prescription dose, mean dose $\left(D_{\text{mean}}\right)$, the dose received by 95% of the volume $\left(D_{95}\right)$, and maximum dose $\left(D_{\text{max}}\right)$ to the PTVs for the XiO, KonRad. and Prowess plans. For most cases, the values for $D_{\text{mean}}$ are similar among the three systems; the differences do not exceed 2.1 Gy.

**Table 2(a):** The prescription dose, mean dose $\left(D_{\text{mean}}\right)$, the dose received by 95% of the volume $\left(D_{95}\right)$, and maximum dose $\left(D_{\text{max}}\right)$ to the PTVs for the XiO, Prowess, and KonRad plans.

*Maximum Dose (Gy)*			$D_{95}$ *(Gy)*			*Mean Dose (Gy)*				
*KonRad*	*Prowess*	*XiO*	*KonRad*	*Prowess*	*XiO*	*KonRad*	*Prowess*	*XiO*	*Prescribed dose (Gy)*	*Tumor Site*
61	58.8	62	51.2	51.3	51.1	53.9	53	52.5	54	Low‐grade Glioma
69.6	71	69	61.6	61.5	62	64.4	66	63.9	64.8	Parotid Carcinoma
53.6	53.9	54	47.8	47.5	47.7	50	51.5	50	50	Pituitary Adenoma
51.62	51.7	52	42.8	42.7	43.1	44.75	46	45	45	Retinoblastoma
73.3	70.7	71.4	61.6	61.5	62	65.1	66.6	65.3	64.8	Parotid Carcinoma
22.3	21.6	22.3	19	18.8	19.1	19.7	20.2	19.8	19.8	High‐risk Medulloblastoma
36.2	35.9	36.4	30.7	30.8	30.9	32.5	33.3	32.3	32.4	Low‐risk Medulloblastoma
41.4	42	41.5	34.4	34.2	34	36.3	38	36.6	36	Nasolaboil RMS
60	62	57.6	47.8	47.8	47.9	50.6	51.8	51	50.4	Rhabdomyosarcoma (RMS)
68.8	65.8	63	56.43	56.4	56.2	59.9	61	59	59.4	Ependymoma
73.5	68.7	69.8	57	57	57.1	59.9	61.6	60	60	Maxillary Sarcoma

Table 3 lists the results of the beam segmentation optimization. The Prowess and KonRad plans show a statistically significant (p−value=0.001) reduction in the number of MUs (between 1.8% and 61.5%) for the different cases. A reduction between 12.5% and 63.5% in the number of beam segments is also noticed.

**Table 2(b):** The dose range, mean percentage of dose, mean percentage of the dose received by 95% of the volume, and mean percentage of maximum dose to the PTVs for the XiO, Prowess, and KonRad plans.

*Dose Range (Gy)*	*Mean Percentage of Dose* $D_{mean}$ *(%)*			*Mean Percentage of* $D_{95}$ *(%)*			*Mean Percentage of Maximum Dose (%)*		
19.8 ‐ 64.8	XiO	Prowess	KonRad	XiO	Prowess	KonRad	XiO	Prowess	KonRad
	99.9	102.4	100.1	95.3	95	95.2	112	112.3	113.8

In this case, as a “post‐hoc” analysis of the group means, it was found that the XiO and Prowess data means differed by 275 units, the XiO and KonRad data means differed by 215 units, and the Prowess and KoRad data means differed by only 60 units. The standard error of each of these differences is 82. Thus the XiO is strongly different from Prowess and KonRad, as the mean difference is more times the standard error. Thus we can be confident that the population mean of the XiO data differs from the population mean of the Prowess and Konrad data. However, there is no evidence that the Prowess and KoRad have different population means from each other, as their mean difference is comparable to the standard error.

Table 4 lists the number of MU/cGy (modulation factor) and the treatment delivery time (sec) for each of the investigated cases. Fogliata et al.(6) determined a mean value of 2.56 MU/cGy for eight TPSs, where KonRad produced the lowest value of 1.9 MU/cGy. Comparable average values are also reported in the present study (2.4 MU/cGy for KonRad, 2.1 MU/cGy for Prowess, and a higher value of 3.6 MU/cGy for XiO).

**Table 3:** Monitor unit per fraction and total segments in each of the studied cases using XiO, Prowess, and KonRad TPSs.

*Total Segments*			*MU/fx*			
*KonRad*	*Prowess*	*XiO*	*KonRad*	*Prowess*	*XiO*	*Tumor Site*
67	35	96	432	284	513	Low‐grade Glioma
51	60	88	355	304	523	Parotid Carcinoma
48	84	79	381	489	548	Pituitary Adenoma
57	72	120	377	463	595	Retinoblastoma
61	48	81	372	411	379	Parotid Carcinoma (carsinoma carcinoma)
71	84	109	481	428	742	High‐risk Medulloblastoma (Posterior fossa)
48	42	114	342	327	724	Low‐risk Medulloblastoma (Posterior fossa)
42	49	78	338	361	591	Nasolaboil RMS
100	98	203	713	374	911	Rhabdomyosarcoma (RMS)
84	42	96	363	292	512	Ependymoma
100	108	237	682	449	1167	Maxillary Sarcoma
66.3	65.6	118	439.6	380	655	Mean
20.5	25	52.8	134.2	72.6	222	SD
	7.864			9.5		F‐test
	0.002			0.001		p‐value

The reduction in the combination of the number of MUs and the number of segments results in significantly shorter (p−value=0.002) delivery times (between 24.7% and 64.5%). as shown in Table 4. The higher number of monitor units (MU), longer delivery time, and higher number of beam segments (delivering higher leakage radiation to the patient) is a disadvantage in XiO compared to Prowess and KonRad. This disadvantage of XiO may be reflected mainly in a possible increase in radiation‐induced secondary malignancies, caused mostly by the increased volume of patient receiving low‐dose levels.^(21)^ Although this issue is not yet certain, it should be taken into consideration in the case of pediatric patients. If true, this problem may represent a major factor limiting the use of XiO in pediatric oncology, knowing that pediatric treatments are delicate where enhanced radiation sensitivity is expected. Children are more sensitive than adults. Moreover, the effect of scattered radiation inside the patient is more significant in the small body of a child than in the large body of an adult. It has been reported that there is a genetic susceptibility of pediatric tissues to radiation‐induced cancer.^(22)^

Table 5 presents the average number of MUs per segment and the number of MUs for the longest and shortest segments in the calculated plans. The main difference between the three systems is that the Prowess plans include segments with very small number of MUs, which may affect the amount of radiation leakage.

**Table 4:** The number of MU/cGy (modulation factor) and the treatment delivery time (sec) using XiO, Prowess, and KonRad TPSs.

*MU/cGy*			*Treatment delivery time (sec)*.			
*KonRad*	*Prowess*	*XiO*	*KonRad*	*Prowess*	*XiO*	*Tumor Site*
2.4	1.6	2.85	291	270	512	Low‐grade Glioma
1.97	2.3	2.91	323	305	646	Parotid Carcinoma
1.9	2.5	2.74	289	281	401	Pituitary Adenoma
2.09	2.6	3.31	320	309	794	Retinoblastoma
2.07	1.7	2.11	368	343	570	Parotid Carcinoma carsinoma carcinoma
2.67	2.4	4.12	450	432	800	High‐risk Medulloblastoma (Posterior fossa)
1.9	1.8	4.02	326	321	620	Low‐risk Medulloblastoma (Posterior fossa)
1.88	2	3.28	302	280	429	Nasolaboil RMS
3.96	2.1	5.06	695	677	1036	Rhabdomyosarcoma (RMS)
2.02	1.6	2.84	483	469	641	Ependymoma
3.41	2.2	5.83	612	598	1687	Maxillary Sarcoma
2.4	2.1	3.6	405.4	389.5	740	Mean
0.7	0.4	1.1	139.2	139	363	SD
	10.87			7.575		F‐test
	0.0003			0.002		p‐value

A feature common to practically all IMRT plans is that a relatively large volume would receive a low dose of radiation. This low‐dose volume may not cause acute or subacute clinical morbidity, but may potentially be carcinogenic, especially in children. Some models of radiation carcinogenesis suggest that the dose‐response relationship is linear up to a dose of 6 Gy, where it then reaches a plateau.^(21)^ The percentage volumes of each patient receiving 2 Gy and 5 Gy may be important in this context. Therefore, the present study reports the percentage volumes of each patient receiving 2 Gy and 5 Gy for comparison between the three TPSs for each treatment plan in Table 6. Results show statistically significant values (p−value=0.0001). As a general trend, KonRad has the highest volume receiving in excess of 2 and 5 Gy, and Prowess has the lowest. Also, KonRad achieves better conformality than either XiO and Prowess, although it is not considered statistically significant.

**Table 5:** The number of MUs per segment, and the number of MUs for the longest and shortest segments, in the calculated plans for XiO, Prowess, and KonRad TPSs.

*Longest Segment (MU)*			*Shortest Segment(MU)*			*MU/Segment*			
*KonRad*	*Prowess*	*XiO*	*KonRad*	*Prowess*	*XiO*	*KonRad*	*Prowess*	*XiO*	*Tumor Site*
14	33	11.3	5.2	1	3.7	6.45	8	5.3	Low‐grade Glioma
14.1	25	14.5	6.2	2	2.9	7	8.6	5.9	Parotid Carcinoma
17	21	16.4	5	1	4.8	7.9	5.8	6.9	Pituitary Adenoma
17	30	13.3	4	1	2.9	6.6	6.4	5	Retinoblastoma
16	16	12.8	5	1	1.1	6.1	5	4.7	Parotid Carcinoma
12.5	17	22.7	5.7	1	3.4	6.8	5.1	6.8	High‐risk Medulloblastoma
14	22	15	6.2	1	3.7	7.1	7.8	6.4	Low‐risk Medulloblastoma
23.9	19	43.3	4.3	2	2.6	8	7.4	7.6	Nasolaboil RMS
13.2	33	13.1	5	1	2	7.1	3.8	4.5	Rhabdomyosarcoma (RMS)
10	21	13.3	3	1	3.4	4.3	7	5.3	Ependymoma
20	16	27.8	5	1	2.1	6.8	4.2	4.9	Maxillary Sarcoma
15.6	23	18.5	4	1.2	3	6.7	6.3	5.8	Mean
3.8	6.4	9.6	1	0.4	1	1	1.6	1	SD

The radiation conformation number of the resultant treatment plans are computed using Eq. (1).^(18)^

This study investigates the usability of the KonRad, Prowess, and XiO TPSs for pathologies which are more complicated in nature, rare, and more challenging (such as pediatric cases) especially in the situation of brain, orbit, and head and neck, where treatment planning requires particular skills and is bounded by dose‐limiting constraints often severely different from the ones applied to adults.

For the 11 cases studied in this work, the three treatment planning systems under comparison allow for the design of plans mostly respecting the initial treatment planning objectives with a range of differences. The plans generate equivalent dose distributions, which are generally expected to correlate with significant reduction of acute and late toxicity, as already documented in pediatric radiation oncology.^(23)^ Each system may provide better capabilities in some clinical requirements and strategies and lower capabilities in others.

**Table 6:** The values of the conformation number and the volumes receiving greater than 2 Gy and greater than 5 Gy in percentage using XiO, Prowess, and KonRad TPSs.

	$V_{\text{receiving}2\text{Gy}}(\%)$			$V_{\text{receiving}5\text{Gy}}(\%)$			*CN*		
*Tumor Site*	*XiO*	*Prowess*	*KonRad*	*XiO*	*Prowess*	*KonRad*	*XiO*	*Prowess*	*KonRad*
Low‐grade Glioma	63	49	90.87	53.14	42.5	69.09	0.7	0.65	0.73
Parotid Ccarcinoma	79.4	46	98.67	68.68	38	78.10	0.68	0.68	0.71
Pituitary Adenoma	84.7	39.4	91.14	71.89	32.4	73.41	0.7	0.75	0.68
Retinoblastoma	90.2	41	81.88	56.86	30.5	48.08	0.69	0.71	0.7
Parotid Carcinoma	73.6	29	79.31	63.08	25	58.26	0.69	0.81	0.71
High‐risk	86.8	14.3	92.17	76.63	12	77.02	0.73	0.74	0.76
Medulloblastoma									
Low‐risk	84.4	11.8	95.86	75.20	9.4	84.03	0.68	0.68	0.69
Medulloblastoma									
Nasolaboil RMS	79.6	24	88.97	47.35	13	53.19	0.7	0.6	0.72
Rhabdomyosarcoma (RMS)	76.9	42.9	74.85	74.71	40.7	69.07	0.68	0.7	0.69
Ependymoma	60.9	71	62.94	54.47	62	53.08	0.71	0.7	0.73
Maxillary Sarcoma	76.4	34.5	80.89	62.64	30	65.12	0.75	0.83	0.75
Mean	77.8	36.6	85	64.1	30.5	66.2	0.7	0.7	0.72
SD	9.3	16.8	10.5	10.1	15.6	11.7	0.02	0.07	0.03
F‐test		47.4			27.4			0.4	
p‐value		0.0001			0.0001			0.59	

**Figure 4:**
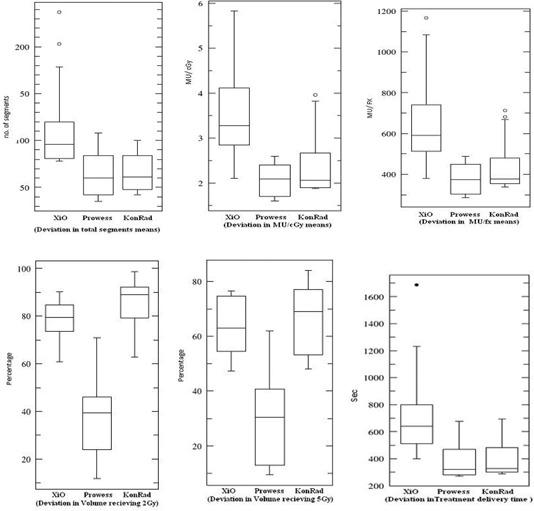
A graphical comparison that provides a visual guide of the deviations in the means between the different groups of data.

In pediatric radiation oncology the number of MU/cGy is a highly important issue in terms of possible induction of secondary malignancies. So the delivered MU to young patients should be as low as possible to minimize that risk. This factor gives an advantage for Prowess, which has a statistically significant (p−value=0.0003) mean modulation factor of 2.1 MU/cGy over KonRad (2.4 MU/cGy) and XiO (3.3 MU/cGy). The variation in means between the different groups of data is shown in (Fig. 4).

This study evaluates, globally, the performances of the three TPSs without explicitly correcting for limitations in dose calculation engines since, in this way, it is possible to reproduce more precisely potential clinical conditions. In addition, it would be substantially impossible to disentangle the optimization phase from the dose calculation engines. In fact, in the XiO TPS, the multileaf segmentation engines include some considerations on scattered radiation from the linac head which are intimately connected with the final dose calculation engines. Therefore, no true factorization process is possible to limit a comparison of performances to the optimization phase.

## IV. CONCLUSIONS

The three treatment planning systems can technically succeed in managing the very restrictive conditions of the clinical goals according to the internationally approved standards and are, in principle, valid for application in pediatric practice. Nevertheless, the XiO TPS shows some drawbacks compared to KonRad and Prowess; the latter two show more favorable results.
